# A High-Efficiency CRISPR–Cas9 Ribonucleoprotein Genome Editing System in *Aspergillus fijiensis* Enabled by Microhomology-Mediated End Joining

**DOI:** 10.3390/jof12030165

**Published:** 2026-02-25

**Authors:** Zhenchun Duan, Shuangfei Zhang, Xueduan Liu

**Affiliations:** 1 School of Minerals Processing and Bioengineering, Central South University, Changsha 410083, China; 2 Key Laboratory of Biohydrometallurgy, Ministry of Education, Changsha 410083, China

**Keywords:** *Aspergillus fijiensis*, genome editing, CRISPR-Cas9, RNP, MMEJ, protoplast transformation, filamentous fungi

## Abstract

*Aspergillus fijiensis* is an industrially important filamentous fungus, whose genetic analysis has been limited by the absence of species-specific tools. This study establishes an optimized CRISPR–Cas9 genome editing platform for *A. fijiensis*, from protoplast preparation to DNA repair pathway engineering. Antibiotic screening first identified hygromycin B and 5-FOA (5-fluoroorotic acid) as effective positive and counter-selection markers. A high-efficiency protoplast regeneration protocol was developed depending on specific osmotic stabilization and mycelial competence. Evaluation of a plasmid-based CRISPR system revealed that while autonomous replication was feasible, gene editing was constrained by low efficiency and a predominant bias toward NHEJ (non-homologous end joining). We implemented a Cas9–sgRNA RNP (ribonucleoprotein) delivery approach, with RNP delivery alone producing frequent indels. However, targeted integration remained inefficient when using conventional MMEJ (Microhomology-mediated end joining) donors. By employing donors containing short (5 bp) microhomology arms between cleavage sites, we effectively engaged the MMEJ pathway, enabling precise insertions and large-fragment deletions in 92% of the analyzed transformants. Donor templates containing minimal 5 bp microhomology sequences could effectively shift the predominant repair pathway from NHEJ to MMEJ. These findings demonstrate that MMEJ is the superior pathway with a unique mechanism for genome engineering in *A. fijiensis*, providing a versatile toolkit for unlocking the biotechnological potential of this recalcitrant species and a successful paradigm for establishing genetic systems in other species.

## 1. Introduction

Filamentous fungi represent a major class of microbial cell factories in the biotechnology industry, largely due to their capacity for high-level protein secretion, flexible secondary metabolism, and efficient utilization of complex carbon sources [1,2,3,4]. In particular, members of the genus *Aspergillus* have been extensively exploited as hosts for large production of all kinds of enzymes, organic acids, and diverse secondary metabolites, underpinning applications in food processing, pharmaceuticals, agriculture, and emerging bio-based industries [5,6,7,8]. Recent advances in fungal genomics and genome mining have further revealed that *Aspergillus* species harbor abundant cryptic biosynthetic gene clusters (BGCs), indicating a vast, largely untapped reservoir of structurally diverse and potentially bioactive compounds [9,10,11,12]. The black aspergilli (section Nigri) constitute an industrially important lineage; however, substantial strain- and species-level diversity within this group often limits the direct transferability of genetic toolkits optimized in a single model chassis [5]. Accordingly, establishing species-adapted genome-editing workflows remains necessary to unlock lineage- and strain-specific metabolic potential and to enable functional interrogation in non-model Nigri members.

Within this genus, *A. fijiensis* has attracted increasing attention as an ecologically and biotechnologically relevant species. Recent studies have reported that an endophytic strain of *A. fijiensis* (GDIZM-1) demonstrates significant potential as a novel biopesticide, causing over 70% mortality in the Asian citrus psyllid (*Diaphorina citri*), a vector of citrus greening disease, with only 3.33% adult survival after 14 days of treatment [13]. Beyond biocontrol, *A. fijiensis* holds substantial industrial value. It is an approved source of safe food enzymes, such as endo-polygalacturonase and endo-1,3(4)-β-glucanase, used in distilled alcohol production, baking, and fruit juice processing [14].The β-fructofuranosidase (fopA) from *A. fijiensis* ATCC 20611 is extensively used for commercial short-chain fructooligosaccharide (scFOS) production, attributed to its high transfructosylating-to-hydrolytic activity ratio and strong regiospecificity on sucrose [15]. Published bioprocess studies have demonstrated that heterologous bioreactor expression of the *A. fijiensis* ATCC 20611 fopA β-fructofuranosidase can reach volumetric activities up to 13,702 U/mL, supporting the feasibility of translating *A. fijiensis*-derived biocatalysts toward scalable manufacturing [15]. Recent studies have also reported that the *Aspergillus* species genome contains multiple predicted polyketide synthase (PKS) and nonribosomal peptide synthetase (NRPS) gene clusters, implying a considerable but underexplored potential capacity for secondary metabolite biosynthesis [9,11,12,16]. However, the functional interrogation of these genetic resources remains severely constrained. A major constraint in exploiting these organisms is the limited availability of efficient, species-specific genome editing tools. For many non-model filamentous fungi, including *A. fijiensis*, genetic manipulation remains technically demanding and labor-intensive, with low success rates. These limitations substantially impede both mechanistic studies of gene function and the rational engineering of industrial strains [5,17,18,19]. Consequently, the development of reliable genome editing strategies tailored to such species is a prerequisite for fully realizing their metabolic and application potential.

Traditionally, genetic modification in filamentous fungi has relied on HDR (Homology-directed repair)-based strategies, typically implemented via polyethylene glycol (PEG)-mediated protoplast transformation and donor DNA constructs flanked by long homology arms [20,21,22]. While reliable in principle, HDR-mediated editing often suffers from low efficiency due to the dominance of the NHEJ pathway, which competes with HDR for the repair of DSB (double-strand breaks), particularly in wild-type genetic backgrounds [23,24,25]. Although disruption of NHEJ components (e.g., *ku70* or *ligD*) can enhance HDR efficiency, such approaches are species-specific and may introduce unintended physiological effects [21,26]. Plasmid-based CRISPR–Cas9 systems have partially alleviated these limitations and are widely used as initial platforms for establishing genetic tractability in non-model fungi [5,27,28,29]. These systems enable stable expression of Cas9 and guide RNAs under defined promoters. Nevertheless, they present intrinsic drawbacks, including prolonged nuclease expression, increased risk of off-target effects, random plasmid integration, and regulatory concerns associated with the retention of foreign DNA sequences [18,30]. In recent years, RNP-based CRISPR delivery—where preassembled Cas9–sgRNA complexes are directly introduced into fungal cells—has emerged as a powerful alternative. RNP-mediated editing offers immediate nuclease activity, transient intracellular presence, and reduced off-target effects, making it particularly attractive for precise and marker-free genome engineering [31,32]. Parallel to this development, MMEJ has been recognized as an efficient DSB repair pathway in fungi. By exploiting short microhomology arms (typically 20–40 bp), MMEJ enables targeted deletions and insertions even in HDR-deficient or NHEJ-dominant backgrounds [3,24,33]. Despite these methodological advances, no genome editing system—neither plasmid-based nor RNP-mediated—has been systematically established or optimized for *A. fijiensis*. Consequently, the efficiency, repair pathway preference, and feasibility of precise gene disruption or large-fragment engineering in this species remain largely unknown. The lack of a standardized genetic toolkit therefore represents a substantial obstacle to functional genomics research and targeted strain improvement in *A. fijiensis*. 

To overcome these limitations, this study was structured around four specific aims: (1) to establish a reproducible, high-yield protoplast preparation and regeneration system for *A. fijiensis*; (2) to construct and assess a conventional plasmid-based HDR editing platform, thereby defining its practical feasibility and limitations; (3) to develop a CRISPR–Cas9 RNP-mediated genome editing strategy that leverages the endogenous MMEJ pathway for gene disruption; and (4) to assess the versatility of the optimized RNP–MMEJ system by performing challenging genome engineering tasks, including large-fragment deletions and targeted insertions. Collectively, these objectives address the central research question of whether an RNP–MMEJ-based framework can overcome species-specific barriers to genome editing in *A. fijiensis* and provide a broadly applicable solution for non-model filamentous fungi.

This work advances the understanding of DNA repair pathway utilization in a previously uncharacterized *Aspergillus* species, providing insight into the feasibility of MMEJ-driven genome editing beyond established model fungi. We present the first systematically optimized genome editing pipeline for *A. fijiensis*, supported by comparative evaluation of plasmid-based and RNP-mediated strategies. The developed RNP–MMEJ toolbox offers a rapid, efficient, and marker-free approach for genome engineering, enabling functional genomics and metabolic engineering of *A. fijiensis* and potentially other recalcitrant filamentous fungi.

## 2. Materials and Methods

### 2.1. Fungal Strain and Culture Conditions

The *A*. *fijiensis* isolate utilized throughout this research was originally obtained from ginkgo roots at our laboratory. Its taxonomic identity was rigorously established via *ITS*, *caM*, and *benA* gene PCR amplification followed by Sanger sequencing verification. Long-term preservation of the strain was maintained in a 25% (*v*/*v*) glycerol solution at an ultra-low temperature of −80 °C. For molecular cloning and plasmid maintenance, *Escherichia coli* DH5α was employed as the host. Fungal cultures were routinely maintained on PDA (Potato Dextrose Agar). Liquid fermentations were conducted in PDB (Potato Dextrose Broth) at 30 °C under constant agitation at 180 rpm. Submerged cultures were initiated by inoculating spores or mycelial fragments into 500 mL baffled Erlenmeyer flasks (Corning, Corning, NY, USA) containing a working volume of 100 mL, and processed on a rotary shaker (ZQZY-78, Zhicheng, Shanghai, China). *E. coli* cells were grown in LB (Luria–Bertani) medium at 37 °C and 180 rpm using identical flask specifications. Preparation of 1000× trace elements and 20× basal salt stock solutions followed previously established protocols, with storage at 4 °C protected from light [34]. Regeneration medium was prepared using 1.2 M sorbitol as osmotic stabilizer and sterilized at 121 °C for 15 min. Appropriate concentrations of hygromycin B or uracil were added aseptically to the medium based on specific screening purposes. To identify suitable selectable and counter-selectable markers for *A*. *fijiensis*, antibiotic sensitivity assays were performed using a mycelial plug method on PDA plates supplemented with commonly used fungal selection agents. In brief, the wild-type isolate was pre-grown on PDA for one week at 30 °C, after which 3 mm diameter agar disks were harvested from the peripheral growth zone using a sterile puncher. These disks were positioned at the center of PDA plates containing antibiotics at both standard and double-strength concentrations. The screening panel included bleomycin (100–200 μg/mL), G418 (100–200 μg/mL), hygromycin B (100–200 μg/mL), phosphinothricin (200–400 μg/mL), and 5-fluoroorotic acid (5-FOA; 1–2 mg/mL). Stock solutions were formulated as per manufacturer guidelines, passed through 0.22 μm filters, and incorporated into the autoclaved medium once it cooled to approximately 50 °C. Following a 7-day incubation at 30 °C, the degree of growth inhibition was quantified by comparing radial colony expansion against drug-free control plates. Complete sensitivity was defined by a total absence of mycelial growth beyond the initial inoculation plug. Basic chemical reagents were sourced from Sinopharm (Shanghai, China), while molecular biology-grade components and enzymes were predominantly procured from Sangon Biotech (Shanghai, China).

### 2.2. Preparation of Mycelia for Protoplast Isolation

To generate germinating spores, the fungus was grown on PDA slants containing sorbitol at 30 °C for a 7-day period. Conidia were liberated using sterile water supplemented with 0.02% (*v*/*v*) Tween-80 and harvested via centrifugation (Avanti J-26S, Beckman Coulter, Brea, CA, USA) at 3000 rpm for 5 min. After two consecutive washes, spore density was adjusted to a concentration of 1 × 10^7^ conidia/mL using a hemocytometer. For the production of germlings, conidia were introduced into fresh PDB medium containing 1% benomyl and agitated at 180 rpm for 8 h at 30 °C. For mature mycelial collection, the incubation duration was varied under the same parameters to optimize protoplast yield. For germling preparation, conidia were inoculated into fresh PDB containing 1% benomyl and incubated at 30 °C and 180 rpm for 8 h in 500 mL baffled shake flasks with a working volume of 100 mL. For mycelial preparation, cultures were incubated for different times under identical conditions prior to protoplast generation. Eight stabilizers were evaluated, including ionic stabilizers (0.7 M NaCl, 0.6 M KCl, 0.6 M MgSO_4_), sugar-alcohol stabilizers (1.2 M mannitol, 1.2 M sorbitol, 1.2 M sucrose), and isotonic buffers (50 mM PBS and 50 mM maleate buffer). Germling-stage mycelia (8 h) were digested in each stabilizer using a moderate enzyme mixture consisting of Lywallzyme (MKbio, MX7356, Shanghai, China) and Yatalase (Takara, T017, Nishinomiya, Japan) for 3 h under otherwise identical conditions. The following enzymes were used: Lywallzyme, Yatalase, cellulase (MKbio, MX7352, Shanghai, China), snailase (MKbio, MF0213, Shanghai, China), lysozyme (MKbio, MF0202, Shanghai, China), and driselase (Sigma, D9515, St. Louis, MO, USA). Six enzyme mixtures were prepared exactly according to Table 1. The mycelial input per digestion was standardized with a wet weight of 0.5 g washed once with 0.7 M NaCl, gently blotted to remove excess liquid, and immediately transferred into enzyme solution. Digestions were performed at 30 °C, 90 rpm for 3 h. For the initial enzyme-combination screening, mycelia harvested at 8 h, 16 h, and 24 h were digested with combinations C1–C6 under otherwise identical conditions. To further refine the protoplast preparation conditions, mycelia harvested at 2 h intervals between 12 and 20 h were digested using enzyme Combination 6 under otherwise identical parameters. After digestion, the suspension was filtered to remove undigested debris. Protoplasts were collected by low-speed centrifugation at 3000 rpm for 10 min at 4 °C, washed gently with 0.7 M NaCl, and resuspended in a defined volume. Protoplast concentration was determined using a hemocytometer. For each biological replicate, protoplasts were counted in at least three independent samples.

### 2.3. Genomic DNA Extraction from A. fijiensis

Isolation of genomic DNA from the fungal biomass was performed using a modified SDS–phenol–chloroform protocol. Mycelia from 3-day-old cultures were harvested through four layers of sterile gauze and rinsed three times with deionized water. The samples were flash-frozen and pulverized into a fine powder under liquid nitrogen, followed by lysis in a specialized buffer (200 mM Tris-HCl pH 8.0, 50 mM EDTA, 2% SDS) at 60 °C for 15 min. To remove lipid and protein contaminants, an equivalent volume of phenol:chloroform:isoamyl alcohol (25:24:1) was added, and the mixture was centrifuged at 12,000× *g* for 15 min. The aqueous phase containing DNA was precipitated using 0.7 volumes of ice-cold isopropanol, cleansed twice with 75% ethanol, and finally dissolved in nuclease-free water. The integrity and concentration of the extracted DNA were verified using a NanoDrop 2000 (Thermo Fisher Scientific, Waltham, MA, USA) and visualized via 1.5% agarose gel electrophoresis.

### 2.4. PCR Amplification, Overlap Extension, and DNA Purification

PCR procedures were carried out using PrimeSTAR Max DNA polymerase (Takara, Nishinomiya, Japan). Each 25 μL reaction volume was composed of 5× PrimeSTAR buffer, 200 μM dNTP mix, 0.4 μM of each specific primer, and 0.5 U of the enzyme. The thermal cycling profile consisted of initial denaturation (98 °C, 2 min), followed by 30 cycles of denaturation (98 °C, 10 s), annealing (56 °C, 15 s), and elongation (72 °C, 1 min), with a final 5 min extension at 72 °C. Analysis of PCR products was conducted on 1% agarose gels. Overlap extension PCR utilized a dual-stage approach: initially, flanking upstream and downstream regions were amplified independently; subsequently, these fragments were fused in a primer-free reaction for 10 cycles using equimolar ratios, followed by exponential amplification using external primers. The resulting DNA fragments were purified with the AxyPrep™ DNA Gel Extraction Kit (Axygen, #AP-GX-250, Tewksbury, MA, USA), as per the provided manual.

### 2.5. Plasmid Construction and E. coli Transformation

For CRISPR–Cas9-mediated *pyrG* gene knockout in *A*. *fijiensis*, the sgRNA expression plasmid and corresponding CRISPR–Cas9 vector were constructed as follows. The sgRNA expression plasmid pPu6-*pyrG*-sgRNA was first generated by amplifying the gRNA scaffold sequence from pX330 plasmid (Addgene, Watertown, MA, USA, #42230) using primers gRNA-scaffold-F and gRNA-scaffold-R (Table S1). PCR amplification was performed with PrimeSTAR Max High-Fidelity DNA Polymerase (Takara, Nishinomiya, Japan), according to the manufacturer’s instructions. The endogenous U6 promoter sequence was amplified from the *A. fijiensis* genome using primers U6-1-F and U6-1-R containing 5’ overhang sequences complementary to the gRNA scaffold. The U6 promoter fragment was fused to the gRNA scaffold using overlap-extension PCR with primers U6-1-F and gRNA-scaffold-R. The resulting fusion fragment was purified using AxyPrep™ DNA Gel Extraction Kit (Axygen, #AP-GX-250, Tewksbury, MA, USA) and cloned into the pEASY-Blunt Zero vector (TransGen Biotech, #CB101-01, Beijng, China) to generate the intermediate plasmid pPu6-sgRNA. The 20 bp *pyrG* target sequence was subsequently inserted between the U6 promoter and gRNA scaffold using seamless cloning with primers U6-*pyrG*-F and U6-1-*pyrG*-R, resulting in the final sgRNA expression plasmid pPu6-*pyrG*-sgRNA. To construct the CRISPR/Cas9 plasmid AFM-Δ*pyrG* for targeted gene knockout, the vector backbone containing the SpCas9 ORF, AMA1 (Autonomous maintenance in *Aspergillus* element 1) autonomous replication element, and flanking linkers was amplified from plasmid FM-6 using primers pAMA-1-F and pAMA-R [34]. The U6-*pyrG*-sgRNA cassette was amplified from pPu6-*pyrG*-sgRNA using primers U6-1-F and gRNA-scaffold-R, and was inserted into the vector backbone through homologous recombination using the ClonExpress® Ultra One Step Cloning Kit (Vazyme, #C115, Nanjing, China) following the manufacturer’s protocol. The resulting pAMA1-Pu6-*pyrG*-sgRNA-PgpdA-Cas9 plasmid (AFM-Δ*pyrG*) was verified by PCR and Sanger sequencing (Sangon Biotech, Shanghai, China) to confirm sequence integrity. This plasmid was subsequently used for protoplast transformation of *A. fijiensis* to achieve *pyrG* gene disruption.

### 2.6. In Vitro Transcription and Purification of sgRNA

Ribonucleoprotein (RNP) complexes were formed by combining purified sgRNA with SpCas9 protein (Synthego, Redwood City, CA, USA) in 1× PBS. The mixture was incubated for 15 min at 37 °C to ensure stable assembly. To assess cleavage efficiency, 1500 ng of target PCR amplicons were incubated with the RNP complexes for 10 min at 37 °C, followed by heat inactivation at 65 °C for 10 min. The resulting DNA fragments were resolved on agarose gels. During assembly, a slight molar excess of sgRNA (1.5:1 ratio of sgRNA to Cas9) was utilized to maximize protein loading. Control experiments omitting either the sgRNA or the RNP complex were performed in parallel to verify that DNA cleavage was specifically mediated by the RNP at the intended locus.

### 2.7. Assembly and In Vitro Validation of Cas9 RNP Complexes

Cas9 RNP complexes were assembled by mixing sgRNA with SpCas9 protein (Synthego, Redwood City, CA, USA) in 1× PBS buffer to a final volume of 20 μL and incubated at 37 °C for 15 min. In vitro cleavage assays were performed using 1500 ng purified PCR amplicons as substrates. Reactions were incubated at 37 °C for 10 mins and terminated at 65 °C for 10 min. Cleavage efficiency was analyzed by agarose gel electrophoresis. Purified sgRNAs were complexed with recombinant SpCas9 protein (Synthego, Redwood City, CA, USA) to form RNPs. SgRNA and SpCas9 were mixed in 1× PBS buffer to a final volume of 20 μL and incubated at 37 °C for 15 min. Unless otherwise specified, sgRNA was supplied in slight molar excess to promote full Cas9 loading (sgRNA:Cas9 = 1.5:1). RNP activity and target specificity were validated using a purified PCR amplicon (~1500 bp) containing the target locus. For each assay, 1500 ng of PCR substrate was incubated with RNPs at 37 °C for 10 min and the reaction was terminated at 65 °C for 10 min. Cleavage products were resolved by 1.5% agarose gel electrophoresis. A no-sgRNA control (Cas9 only) and no-RNP control (substrate only) were included. No detectable cleavage in controls was taken as evidence of sgRNA-dependent target specificity.

### 2.8. Protoplast Preparation and PEG-Mediated Transformation

Mycelia harvested after 18 h of growth were rinsed with 0.7 M NaCl and subjected to enzymatic digestion in a 0.7 M NaCl solution at 30 °C (90 rpm) for a duration of 4 h. The liberated protoplasts were separated from debris using sterile gauze and concentrated by centrifugation at 5000 rpm for 10 min (4 °C). The protoplast pellets underwent two washing steps with STC buffer (1.2 M sorbitol, 10 mM Tris-HCl pH 7.5, 50 mM CaCl_2_) before being resuspended to a final density of 1 × 10^9^ cells/mL for PEG-mediated transformation. For each transformation, 80 μL of protoplast suspension was mixed with pre-assembled Cas9 RNP complexes and, when applicable, 5 ug donor DNA. The mixture was incubated on ice for 30 min, then adding PEG solution supplemented with Triton X-100 (0.01%). After incubation at 37 °C for 30 min, protoplasts were added 1 mL of STC buffer and mixed gently with molten 0.6–0.8% agar SMM containing the appropriate selection agent. The mixture was poured onto a pre-solidified 2.0% agar SMM solid medium with the same osmotic stabilizer and selection agent concentration. The basal SMM regeneration medium contained. For selectable-marker integration experiments, hygromycin B was added to a final working concentration of 100 μg/mL. For *pyrG*-based selection, uracil and uridine were supplemented. Plates were incubated at 30 °C for 7 days.

To determine the dosage dependence of RNP-mediated editing, RNPs were delivered across a final concentration range of 20–300 nM using otherwise identical transformation conditions. Reactions lacking RNPs served as negative controls to evaluate background survival and donor-independent resistance.

### 2.9. Phenotypic Assessment of Edited Strains

Transformants were incubated at 30 °C for 7 days. Putative colonies were subcultured, and genomic DNA was extracted for diagnostic PCR and Sanger sequencing to confirm target site mutations. For *pyrG* disruption, PCR products spanning the predicted cleavage site were subjected to Sanger sequencing to identify indels, frameshifts, or truncating mutations. Phenotypes were evaluated by growth assays on defined media with/without uracil/uridine supplementation. Briefly, verified *pyrG* mutant candidates were point-inoculated onto minimal medium plates supplemented with uracil/uridine (Ura/Uri+) and in parallel onto plates lacking uracil/uridine (Ura/Uri−), then incubated at 30 °C for 7 days. Loss-of-function *pyrG* mutants were scored as auxotrophs showing robust growth on Ura/Uri+ plates but impaired/no growth on Ura/Uri− plates. Where applicable, resistance-based phenotypes were scored on PDA/SMM plates containing hygromycin B (100 μg/mL) relative to no-antibiotic controls. For donor-dependent integration experiments, diagnostic junction PCRs (5′ and 3′ junctions) were performed using primer pairs binding outside the donor homology region and within the selectable marker cassette. Verified mutants were purified by single-colony isolation and stored as glycerol stocks for further analysis. 

### 2.10. Data Processing and Statistical Analysis

The experimental data are expressed as the mean value ± standard deviation (SD) derived from a minimum of three biological replicates. To assess statistical differences, we applied one-way analysis of variance (ANOVA) followed by Tukey’s post hoc test for pairwise comparisons. A *p*-value threshold of <0.05 was established for statistical significance. All computational analyses and graphical renderings were performed using GraphPad Prism version 9.0.

## 3. Results

### 3.1. Strain Verification and Selection Marker Identification

To confirm the taxonomic identity of the strain used throughout this study, we sequenced three commonly used *Aspergillus* barcoding loci (*ITS*, *caM*, and *benA*) and compared them with reference sequences from *A. fijiensis* CBS 313.89, *Aspergillus aculeatus* CBS 172.66, and *Aspergillus aculeatinus* CBS 1212060. ClustalW alignments showed that the experimental isolate displayed the highest sequence similarity to *A. fijiensis* across all three markers (Figure S1a). Consistently, maximum-likelihood phylogenetic analysis based on the concatenated *ITS–caM–benA* dataset placed the isolate robustly within the *A. fijiensis* clade, clearly separated from *A. aculeatinus* and *A. aculeatus* (Figure S1b). These results verify that the experimental strain is *A. fijiensis*, providing a validated basis for downstream optimization of selection conditions and genome editing. 

**Figure 1:**
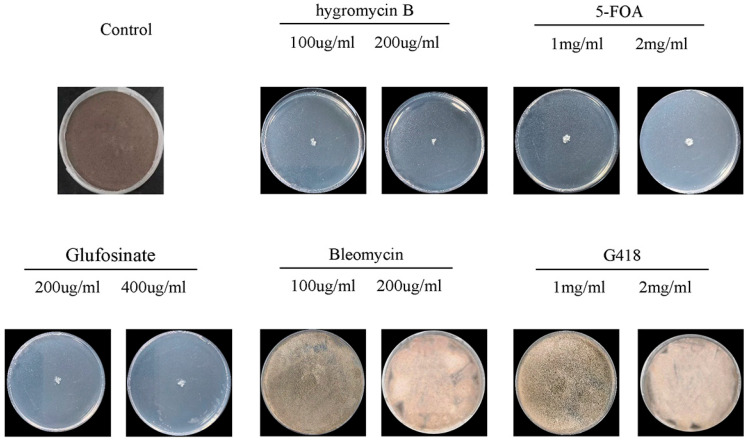
Marker screening for *A. fijiensis*. Sensitivity of the wild-type (WT) strain to different selectable agents with standard concentration and double was evaluated on solid medium. Plates were arranged as follows: WT control, hygromycin B (100 and 200 μg/mL), 5-fluoroorotic acid (5-FOA; 1 and 2 mg/mL), glufosinate (200 and 400 μg/mL), bleomycin (100 and 200 μg/mL), and G418 (1 and 2 mg/mL). All plates were incubated at 30 °C for 7 days.

We evaluated the antibiotic sensitivity profile of the wild-type strain to identify suitable selectable and counter-selectable markers. To this end, *A. fijiensis* was cultured on agar plates supplemented with commonly used fungal selection agents at both standard and double standard concentrations. The wild-type strain displayed complete tolerance to bleomycin and G418 at concentrations of 100 and 200 μg/mL (Figure 1), indicating intrinsic resistance to these antibiotics. Consistent growth was observed even at doubled concentrations, suggesting that neither bleomycin nor G418 is suitable as a positive selection marker in *A. fijiensis*. In contrast, *A. fijiensis* was highly sensitive to hygromycin B, with complete inhibition of colony formation at both 100 μg/mL and 200 μg/mL. Similarly, exposure to 1 mg/mL and 2 mg/mL 5-fluoroorotic acid (5-FOA) effectively suppressed fungal growth, confirming an intact pyrimidine biosynthesis pathway and validating the use of *pyrG*-based counter-selection in *A. fijiensis*. Although phosphinothricin displayed strong antifungal activity at 200 and 400 μg/mL, it was not adopted for subsequent transformation experiments due to considerations of selection stringency and experimental compatibility, as well as its potential cytotoxicity during protoplast regeneration. Based on these results, we selected *hph* (*Hygromycin B phosphotransferase*) as the positive selection marker and 5-FOA (*pyrG*) as the counter-selectable agent for all subsequent genetic manipulations.

### 3.2. Establishment of a High-Efficiency Protoplast Preparation System in A. fijiensis

**Figure 2:**
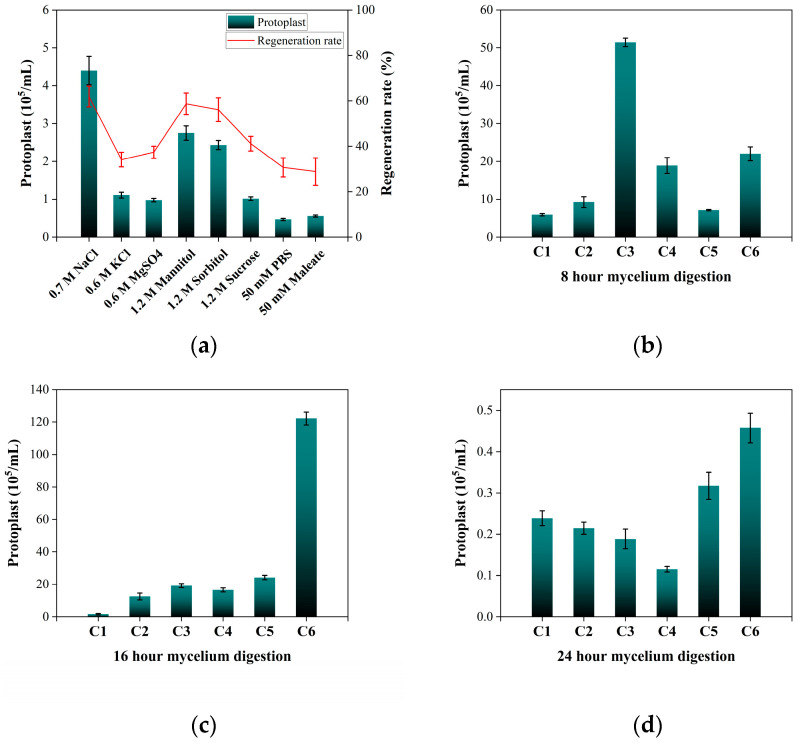

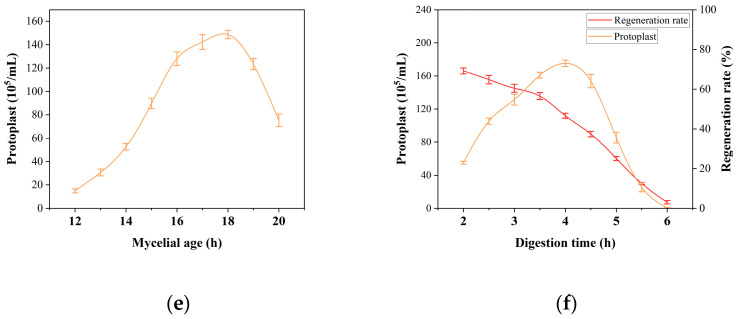
Optimization of protoplast preparation conditions for *A. fijiensis*. (**a**) Stability of *A. fijiensis* protoplasts under different osmotic stabilizers during enzymatic digestion. Eight stabilizers were evaluated, including NaCl, KCl, MgSO_4_, mannitol, sorbitol, sucrose, phosphate buffer, and maleate buffer. (**b**) Effects of different enzymatic combinations on protoplast yield from 8 h mycelia. (**c**) Effects of different enzymatic combinations on protoplast yield from 16 h mycelia. (**d**) Effects of different enzymatic combinations on protoplast yield from 24 h mycelia. (**e**) Fine-scale optimization of mycelial culture time for protoplast production using enzyme Combination 6, with mycelia harvested at 2 h intervals between 12 and 20 h. (**f**) Effect of enzymatic digestion duration on protoplast yield using 18 h mycelia and enzyme Combination 6. Protoplast yields differed significantly among the tested conditions (one-way ANOVA, *p* < 0.05).

The establishment of a reliable protoplast-based transformation system is a prerequisite for genetic manipulation in *A*. *fijiensis*, as no genetic transformation protocol has previously been reported for this species. Because unstable osmotic conditions would cause extensive protoplast rupture and render yield evaluation impossible, we first performed a stabilizer screen prior to any enzyme or growth-stage optimization. *A. fijiensis* protoplasts exhibited markedly different stability across these conditions. Only mannitol, sorbitol, and NaCl supported intact spherical protoplasts throughout the digestion and purification process, whereas MgSO_4_, KCl, Sucrose, phosphate buffer, and malate buffer caused rapid bursting or deformation, preventing reliable quantification (Figure 2a). One-way ANOVA revealed that the choice of osmotic stabilizer had a significant effect on protoplast recovery, F(7, 16) = 113.8, *p* < 0.001. Post hoc comparisons indicated that protoplasts generated under the 0.7 M NaCl conditions achieved a significantly higher maximum yield of 4.40 ± 0.38 × 10^5^ protoplasts/mL compared to all other stabilizers, including 1.2 M mannitol 2.75 ± 0.19 × 10^5^ protoplasts/mL and 1.2 M sorbitol 2.43 ± 0.12 × 10^5^ protoplasts/mL (*p* < 0.05). These protoplasts regenerated efficiently on regeneration medium, producing morphologically normal colonies (Figure S2). Regeneration rates exceeded 65%, confirming that the digestion conditions did not compromise physiological viability. Based on stability and recovery consistency, 0.7 M NaCl was selected as the working osmotic stabilizer for all subsequent optimization steps.

Following stabilizer selection, the effects of mycelial age and enzymatic digestion combinations on protoplast yield were evaluated. Because commercial lysing enzymes traditionally used for fungal protoplast preparation are no longer available, Lywallzyme was employed as a substitute lytic component in combination. Across all digestion treatments, protoplast release exhibited a strong dependence on mycelial age. *A. fijiensis* produced high protoplast yields at two distinct growth phases: (i) early exponential-phase mycelia (~8 h) and (ii) mid-log phase mycelia (~16 h). For 8 h cultures, enzyme combination 3 yielded 5.14 ± 0.11 × 10^6^ protoplasts/mL (Figure 2b), suggesting that at ~8 h the nascent cell wall—still relatively thin and weakly cross-linked—can be efficiently disrupted without requiring additional polysaccharidases. In contrast, 16 h mycelia required a broader-activity enzyme combination 6 containing snailase and lysozyme in addition to lysing enzymes to digest more mature, structurally reinforced walls (Figure 2c). Conversely, at 24 h, mycelia presented negligible protoplasts (approximately 4.57 ± 0.36 × 10^4^ protoplasts/mL), largely because their mature cell walls were highly resistant to degradation (Figure 2d). Statistical analysis confirmed that the enzyme combination significantly influenced the outcome, F(5, 12) = 874.2, *p* < 0.001, with Combination 6 producing a significantly higher number of protoplasts than all other combinations at the 16 h time point (*p* < 0.001), reaching a maximum yield of 1.22 ± 0.04 × 10^7^ protoplasts/mL. The performance ranking indicates that simply adding a single auxiliary enzyme was insufficient, whereas cocktails integrating both “broad-spectrum polysaccharidases” and “wall-loosening/auxiliary activities” provided the most robust and reproducible improvement, particularly for the 16 h condition. These results demonstrate that minimal lytic pairing is adequate for early-phase mycelia, whereas multi-activity cocktails are necessary to efficiently digest mature cell walls.

Based on the superior performance of Combination 6 in the preliminary screening, a second round of optimization was conducted to further refine the mycelial age. This time-course analysis showed a significant main effect of mycelial age on yield, F(8, 18) = 194.5, *p* < 0.001, and identified 18 h mycelia as optimal, yielding approximately 1.49 ± 0.04 × 10^7^ protoplasts/mL (Figure 2e). According to the multiple comparison test, the yield at 18 h was significantly greater than those of both younger (<14 h) and older (>20 h) mycelia, *p* < 0.05. suggesting that cell wall architecture during mid-logarithmic growth critically determines enzymatic susceptibility. Overall, the quantitative comparison demonstrates that optimal protoplast generation requires both an appropriate enzymatic cocktail and a suitable mycelial developmental stage, with the highest efficiency achieved using 18 h mycelia digested with Combination 6. Digestion duration was likewise a key determinant of outcome. One-way ANOVA showed that incubation time significantly affected yield, F(8, 18) = 485.6, *p* < 0.001. Protoplast release increased rapidly and peaked at 4 h, reaching 1.75 ± 0.04 × 10^7^ protoplasts/mL (Figure 2f). Post hoc analysis indicated that the yield at 4 h was significantly higher than those at all other time points, *p* < 0.05, whereas prolonged digestion (>5 h) caused a marked decline, with yields dropping to 2.54 ± 0.55 × 10^6^ at 5.5 h. The regeneration test showed a continuous decrease in recovery rates with increasing digestion time, representing a trade-off between protoplast yield and viability. The 4 h digestion time reached an optimal balance point when maximal release is achieved without compromising regenerative capacity. 

Collectively, these results establish a reliable and efficient protoplast preparation system for *A. fijiensis*, characterized by NaCl-based osmotic stabilization, mid-log-phase mycelia, a broad-spectrum enzymatic cocktail, and a carefully defined digestion window. This system provides a solid foundation for high-efficiency protoplast-mediated transformation and subsequent functional genetic studies in this previously intractable species.

### 3.3. Construction and Functional Assessment of an AMA1-Based Replicative Plasmid System in A. fijiensis

**Figure 3:**
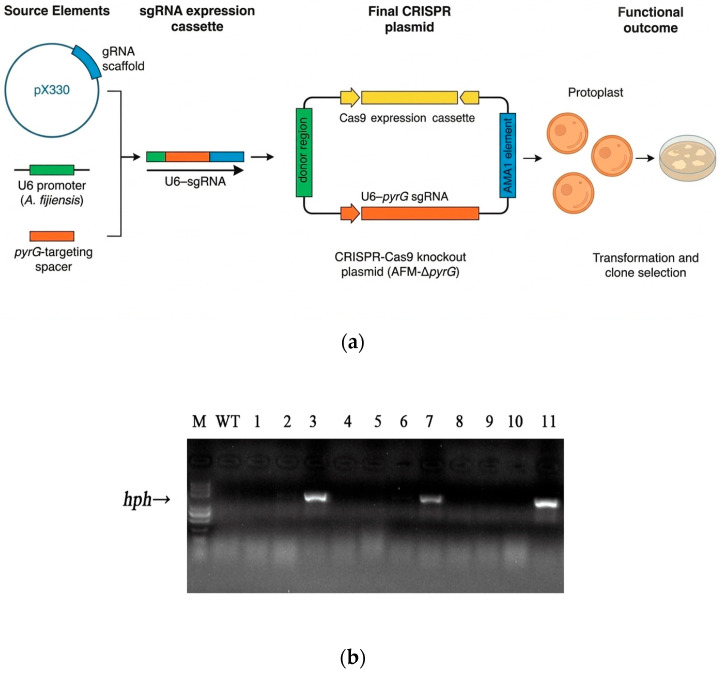
Results of CRISPR–Cas9 plasmids for targeted gene disruption in *A. fijiensis*. (**a**) The sgRNA scaffold sequence was amplified from plasmid pX330 using primers gRNA-scaffold-F and gRNA-scaffold-R. The predicted endogenous U6 promoter was amplified from the *A. fijiensis* genome using adaptor primers U6-1-F and U6-1-R containing 5′ overlapping sequences of the sgRNA scaffold. the 20 bp *pyrG*-targeting sgRNA sequence was seamlessly inserted between the U6 promoter and sgRNA scaffold using primers U6-*pyrG*-F and U6-1-*pyrG*-R. The plasmid backbone containing the Cas9 open reading frame and the AMA1 autonomous replication element was amplified from plasmid FM-6 using primers pAMA-1-F and pAMA1-R. The U6-*pyrG*-sgRNA expression cassette was amplified from pPu6-*pyrG*-sgRNA using primers U6-1-F and gRNA-scaffold-R, and subsequently inserted into the Cas9-containing backbone by homologous recombination to generate the final plasmid AFM-Δ*pyrG*. For clarity, DNA elements in the schematic are not drawn to scale. (**b**) Targeted disruption of the *pyrG* gene mediated by plasmid-based CRISPR-Cas9 editing in *A. fijiensis*. Diagnostic PCR analysis of genomic DNA from independent transformants in *pyrG* locus. The DNA molecular weight marker used was a 100–5000 bp DNA Marker III (Biosharp BL103A, Anhui, China). PCR amplification was performed using primers flanking the targeted integration region to distinguish wild-type and disrupted alleles.

To establish a reusable plasmid-based genetic manipulation platform for *A*. *fijiensis*, an AMA1-dependent autonomous vector was constructed that integrates three functional modules: (i) a codon-optimized *SpCas9* driven by the constitutive *PgpdA* promoter, (ii) an sgRNA expression cassette composed of the predicted endogenous *A. fijiensis* U6 promoter fused to the canonical gRNA scaffold, and (iii) a homologous recombination donor unit targeting the *pyrG* locus. This modular design was intended to assess whether *A. fijiensis* supports U6-driven guide RNA expression, sustained Cas9 production, and stable AMA1-mediated episomal maintenance—prerequisites for plasmid-based genome engineering and promoter validation. The sgRNA expression cassette was generated by fusing the predicted *A. fijiensis* U6 promoter to the conserved gRNA scaffold sequence, and a 20 bp *pyrG*-targeting spacer was subsequently introduced by seamless cloning. This cassette was assembled together with the *PgpdA*-Cas9 module and the AMA1 replicator into a single autonomous plasmid (AFM-Δ*pyrG*) (Figure 3a). Following PEG-mediated transformation into *A. fijiensis* protoplasts, dozens of hygromycin-resistant colonies were obtained, exhibiting stable growth and uniform morphology. Plasmid maintenance assays revealed that over 80% of transformants retained the AMA1-based plasmid after five successive passages under selection, demonstrating robust episomal replication in *A. fijiensis*. This stability is comparable to previously reported AMA1 systems in *Aspergillus nidulans* and *Aspergillus fumigatus*, confirming functional conservation of AMA1-dependent replication in this species.

Using a 1.5 kb donor cassette flanking the *pyrG* locus, plasmid-mediated HDR resulted in targeted gene disruption efficiencies of 27.2%, as determined by diagnostic PCR screening of independent transformants (Figure 3b). These efficiencies are consistent with the lower range reported for AMA1-based CRISPR systems in filamentous fungi, indicating that the endogenous U6 promoter is competent for sgRNA expression and that Cas9 is functionally expressed in *A. fijiensis*. Despite optimization of donor length and promoter configurations, HDR frequencies did not increase substantially beyond this range, suggesting an intrinsic ceiling for plasmid-mediated genome editing in this organism. Collectively, these results demonstrate that while the AMA1-based plasmid system provides a reliable platform for stable gene expression, promoter evaluation, and genetic toolkit development, its capacity for high-efficiency genome editing is limited, thereby motivating the exploration of alternative editing strategies.

### 3.4. Establishment of an Efficient CRISPR-Cas9 RNP Editing Platform in A. fijiensis

**Figure 4:**
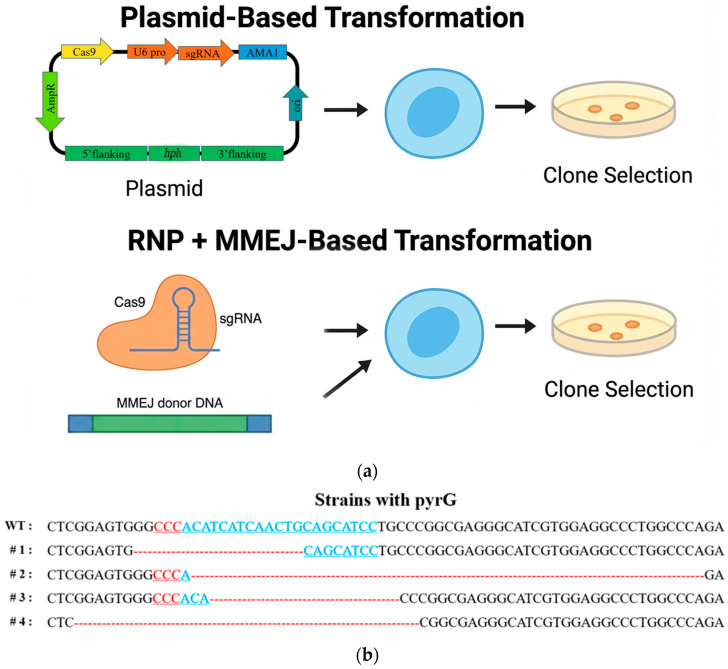
(**a**) Comparison of plasmid-based HDR and RNP + MMEJ-based genome editing strategies in *A. fijiensis*. Plasmid-based genome editing was performed by protoplast-mediated transformation using an AMA1-based CRISPR–Cas9 vector carrying a donor cassette in which the *hph* gene was flanked by 5′ and 3′ homology arms, including a Cas9 coding cassette and sgRNA expression cassette driven by an endogenous U6 promoter. Following Cas9-induced double-strand breaks (DSBs), precise integration of the donor cassette occurred predominantly through HDR. In contrast, RNP-mediated genome editing was conducted by delivering preassembled Cas9–sgRNA ribonucleoprotein complexes together with a linear donor fragment containing short microhomology regions. (**b**) Sequence profiles of representative *pyrG* knockout mutant strains. Aligned sequences spanning the CRISPR–Cas9 target site are shown for selected mutant clones. Blue and red letters indicate target sequence and PAM (protospacer adjacent motif) sequences, red bar indicates deleted nucleotides.

The preliminary evaluation of the plasmid system demonstrated that *A. fijiensis* possesses an extremely rapid NHEJ repair response activity, resulting in limited editing efficiency and possible ectopic donor integration when Cas9 is constitutively expressed. Therefore, we developed and optimized a CRISPR-Cas9 RNP platform using purified recombinant SpCas9 complexed with in vitro-transcribed sgRNAs (Figure 4a). All sgRNAs were synthesized by T7-driven transcription and purified to remove abortive products. In vitro cleavage assays using a 1500 bp PCR amplicon containing the target site demonstrated rapid and dose-dependent DNA cleavage within 10 min, confirming high intrinsic activity of the assembled RNP complexes (Figure S3). No detectable cleavage was observed in control reactions lacking sgRNA, verifying target specificity.

We first investigated the effect of Cas9 RNP concentration on genome editing efficiency in *A. fijiensis*. The *pyrG* locus was selected as a phenotypic knockout target, and RNP complexes were delivered over a concentration range of 20–300 nM using PEG-mediated protoplast transformation (Table 2). No transformants were recovered from reactions lacking RNPs or containing ≤20 nM RNP, indicating that spontaneous background resistance or donor-independent survival was negligible under the conditions tested. As the RNP concentration increased, the total number of recovered CFU (colony-forming units) increased in a clear dose-dependent manner. At intermediate concentrations (60–100 nM), only a small number of transformants were obtained, whereas a marked increase in CFUs was observed at ≥150 nM RNP. Genotypic analysis of representative transformants revealed that the proportion of correctly edited clones increased in parallel with total colony numbers. At 200 nM RNP concentration, sequencing of the *pyrG* locus in a total of 55 transformants showed that 43 clones (78%) carried frameshift or truncating mutations in the vicinity of the predicted Cas9 cleavage site, substantially exceeding knockout efficiencies achieved using plasmid systems at the same locus (Figure 4b). These results demonstrate that RNP-mediated editing in *A. fijiensis* is strongly dosage-dependent and capable of supporting highly efficient gene disruption in the absence of donor templates. To further enhance transformation efficiency, parameters affecting protoplast membrane permeability were systematically optimized. Among the conditions tested, the addition of a low concentration of Triton X-100 during PEG-mediated transformation significantly increased the number of recovered transformants. Compared with PEG treatment alone, inclusion of Triton X-100 resulted in an approximately 1.5-fold increase in CFUs without detectable loss of protoplast viability (Table S2). Based on these results, 55% PEG3350 supplemented with Triton X-100 was selected as the standard condition for subsequent RNP-mediated editing experiments.

This result confirms that RNP-mediated editing can achieve highly efficient gene disruption when combined with appropriate transformation protocol. While single-gene disruption was readily achieved, it remained unclear whether such efficiency could be extended to donor-dependent genome modifications requiring precise DNA integration. To address this limitation, we next evaluated whether RNP-mediated cleavage alone is sufficient to support targeted insertion of exogenous DNA. Donor templates carrying the hygromycin resistance gene (*hph*) with 40 bp flanking sequence microhomology at the Cas9 cleavage junction were first tested under otherwise identical transformation conditions. Genotypic screening of 84 independent transformants failed to identify any clones with correctly insertion; instead, repair outcomes were dominated by small indels or non-specific integration events. These results indicate that a donor with MHS (Microhomology sequence) is not sufficient for productive insertion in *A. fijiensis*, revealing a critical requirement for pathway-specific repair cues. We designed donor templates containing 5 bp MHS around the Cas9 cleavage site, flanked by 35 bp outward extensions. This strategy specifically isolates the function of true MHS identity (the 5 bp fully identical sequence) from the influence of extended flanking homology. Surprisingly, the outcomes were dominated by precise MMEJ-mediated junctions with editing efficiencies of 92%. Donors lacking the 5 bp identical MHS on both sides of the cutting site completely lost insertion efficiency, confirming that true microhomology—not flanking similarity—is the essential determinant. These findings experimentally verify that MMEJ and NHEJ compete during repair in *A. fijiensis*, but the presence of a perfect 5 bp MHS strongly biases the pathway toward MMEJ. Taken together, the data reveal a clear repair preference hierarchy: MMEJ > NHEJ > HDR. This supports the rational design of high-efficiency, small-homology donor cassettes for rapid marker-free genome editing.

### 3.5. Dissecting Microhomology-Mediated End Joining (MMEJ) Repair Characteristics and Applications in A. fijiensis

**Figure 5:**
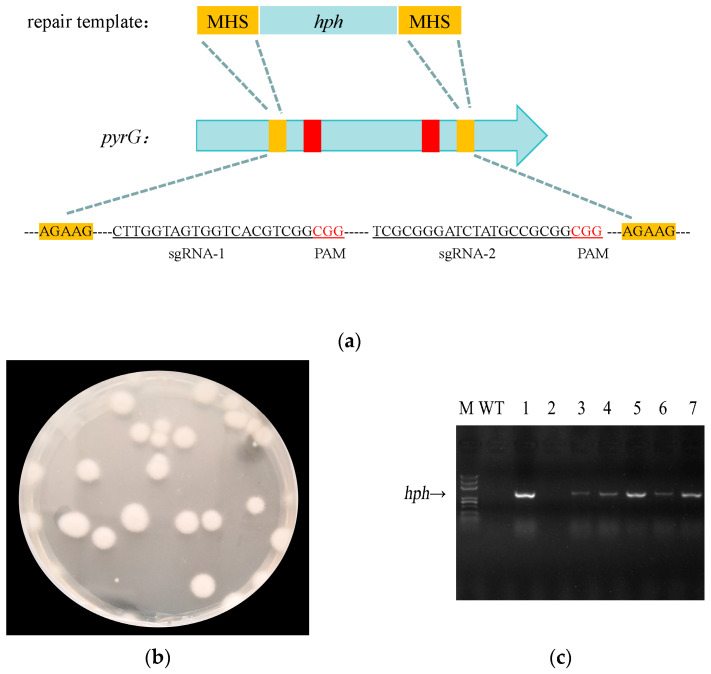
MMEJ-mediated targeted disruption of the *hph* marker gene in *A. fijiensis* using an RNP-based system. (**a**) Schematic illustration of the Cas9 cleavage pattern and microhomology-mediated end joining (MMEJ) repair strategy at the *hph* locus. Two Cas9 target sites flanking the *hph* coding region were designed, each associated with a pair of MHSs (highlighted in yellow) positioned adjacent to the predicted double-strand break sites. The sgRNA protospacer sequences are underlined, and the corresponding PAM motifs are shown in red and underlined. (**b**) Representative mutant clones generated by RNP-mediated editing. (**c**) PCR screening of *hph* insertion events in independent transformants. Molecular weights of DNA fragments were determined using a 100–5000 bp DNA Marker III (Biosharp BL103A, Hefei, Anhui, China). Diagnostic amplification using primers flanking the *hph* locus distinguishes wild-type alleles from MMEJ-mediated deletion alleles by size differences in the PCR products. Positive clones exhibiting the expected bands are indicated.

Having established microhomology as the key factor enabling controlled DNA insertion at a single locus, we next assessed whether this MMEJ-assisted RNP strategy could support more complex genome modifications involving coordinated repair of two double-strand breaks. Donor templates were therefore designed to replace the genomic fragment between two Cas9 cleavage sites (Figure 5a). Using MHS-flanked donors, precise fragment replacement was achieved in 84 of 91 (92%) randomly selected transformants (Figure 5b,c). These results demonstrate that MMEJ-assisted RNP editing is not limited to simple junction repair but can efficiently mediate long-fragment replacement across dual target sites. To define the minimal microhomology requirement for efficient MMEJ-mediated insertion, donor templates carrying 3, 5, or 7 bp perfectly matched microhomology sequences flanking the Cas9 cleavage site were evaluated. Genotypic screening revealed a strong dependence of targeted insertion on microhomology length. Donors containing 3 bp microhomology yielded only sporadic and unstable insertion events (7 of 92 analyzed transformants, 7.6%) (Table 3), whereas increasing the microhomology length to 5 bp resulted in a sharp increase in precise on-target integration (17 of 85 clones, 20%). Further extension of microhomology to 7 bp produced no gains in overall insertion frequency, with correct integration detected in 11 of 87 (12.6%) transformants. In particular, further extension to 7 bp altered the distribution of junction positions, which could potentially be responsible for the decrease in insertion frequency, indicating that both the length and lcous of microhomology impose positional constraints on MMEJ-mediated repair. To assess how flanking homology beyond the core microhomology contributes to integration robustness, additional donor templates were designed in which short homology arms were extended outward from each microhomology region. Outward extensions of 15–35 bp improved junction uniformity and stability, but did not rescue productive insertion when the central microhomology was absent. These results indicate that a short, correctly positioned microhomology is the primary determinant for MMEJ initiation in *A. fijiensis*, whereas flanking homology functions as a mainly stabilizing element that significantly enhances junction quality. 

Together, these results establish a highly efficient, precise RNP-based CRISPR–Cas9 system for *A. fijiensis*. Compared with plasmid-based methods, the RNP platform provides substantially improved editing rates and superior compatibility with MMEJ-assisted repair. This mechanistic insight provides a rational basis for the design of compact, marker-efficient donor cassettes, making it the preferred approach for generating both gene knock-outs and knock-ins in filamentous fungi.

## 4. Discussion

The lack of a dedicated genetic toolkit has long hindered the exploration of *A. fijiensis*, an industrially relevant fungus with significant biocontrol and metabolic potential. By sequentially benchmarking plasmid-based HDR and developing an RNP-mediated CRISPR–Cas9 system coupled with the endogenous MMEJ pathway, we addressed long-standing technical bottlenecks that have constrained functional genomics in this species. Our findings not only provide a validated pipeline for genome engineering but also offer mechanistic insights into the DNA repair preferences of this species; specifically, the critical role of microhomology in bypassing the dominant NHEJ pathway. 

A fundamental prerequisite for PEG-mediated transformation in filamentous fungi is the preparation of highly viable and transformation-competent protoplasts. In this study, systematic optimization of mycelial age, enzymatic digestion conditions, and osmotic stabilizers enabled the consistent production of 1.7 × 10^7^ protoplasts per mL from *A. fijiensis*, providing a robust foundation for subsequent genome-editing experiments. While de Bekker et al. [35] demonstrated the efficacy of specific enzyme cocktails in *Aspergillus niger*, our results indicate that *A. fijiensis* requires distinct enzymatic ratios and osmotic support to overcome cell wall recalcitrance, where protoplast yield proved highly dependent on a narrow developmental window of the mycelia, underscoring the particular sensitivity of *A. fijiensis* to cell wall remodeling dynamics. This sensitivity likely reflects rapid shifts in cell wall cross-linking dynamics during the exponential phase, reinforcing the concept that “standard” *Aspergillus* protocols cannot be applied indiscriminately to non-model relatives [36]. This species-specific requirement is also evident when benchmarking protoplast yields reported for other *Aspergillus* systems. A systematic PMT toolbox study in aconidial industrial *A. niger* strains found that the choice of mycelium pre-culture medium substantially shifted recoverable protoplast numbers; among CPZ-old, MM1, and MM2, CPZ-old supported the highest output, reaching ~1.69 × 10^7^ protoplasts/mL [37]. In an RNP-oriented CRISPR workflow developed for *Aspergillus oryzae*, optimization of the digestion step using a defined compound enzyme mixture produced approximately 5.2 × 10^6^ protoplasts per mL, highlighting that even in a well-studied industrial species, yield is strongly constrained by enzyme composition and digestion design [38]. Our optimized *A. fijiensis* protocol achieved a maximum yield of 1.7 × 10^7^ protoplasts/mL, matching the upper range reported for industrial *A. niger* under its best-performing conditions. Collectively, these comparisons emphasize that “high yield” in *Aspergillus* is operationally species- and protocol-specific, and that *A. fijiensis*—despite being non-model—can reach elite protoplast productivity once the developmental window and enzyme/osmotic parameters are tuned.

Using an AMA1-based plasmid system expressing Cas9 and sgRNA, we confirmed that *A. fijiensis* supports episomal maintenance, heterologous promoter activity, and stable Cas9 expression reaching 27.2% HDR efficiency. Nevertheless, the repair outcome was still overwhelmingly NHEJ-driven, and the functional presence of Cas9–sgRNA did not translate into predictable, high-frequency locus replacement. This finding is consistent with reports in Aspergilli [39] and *Trichoderma reesei* [40], where plasmid-based systems frequently suffer from low targeting efficiency (~1–5%) unless NHEJ components (e.g., *ku70* or *ku80*) are genetically disabled [41,42]. In other filamentous fungi and *Aspergillus* systems where donor-assisted editing succeeds, reported outcomes still vary widely by species and design; for example, HDR-dependent knockouts in *A. niger* have been summarized at ~75–80% in some loci/strains, yet can drop to ~40.9% for albA under otherwise standard PMT/HR conditions [37,43]. In *A. oryzae*, NHEJ-type mutagenesis at niaD typically reached only ~10–20%, and wA disruption showed extreme sgRNA-dependence ranging from 0% to ~68% across guides/transformants, underscoring strong performance variability even when Cas9 expression is achieved [22]. Consistent with reports on AMA1-based vectors in *A. oryzae*, we observed that episomal maintenance facilitates Cas9 expression but does not override the strong endogenous preference for NHEJ in the absence of DNA repair pathway engineering [22]. Moreover, episomal systems can be operationally constrained by plasmid stability/curing dynamics (e.g., truncated AMA1 designs reported plasmid loss on the order of ~33% in *Aspergillus*), which further contributes to heterogeneous Cas9 dosage and reduces the consistency and throughput of downstream phenotypic screening and genotyping [44]. More practically, our findings highlight an often-understated limitation of plasmid-based CRISPR systems: even when functional, their construction workload, variable performance across transformants, and limited throughput make them suboptimal for rapid functional genomics in non-model fungi. In *A. fijiensis*, these constraints are exacerbated by highly efficient NHEJ, rendering conventional HDR strategies impractical for routine use.

Delivery of pre-assembled Cas9–sgRNA RNP complexes allows for targeted DNA cleavage without introducing exogenous genetic material, eliminating concerns associated with plasmid integration and prolonged Cas9 expression, a strategy successfully employed in *A. fumigatus* [34], *Penicillium chrysogenum* [21], and *Botrytis cinerea* [45]. However, our experiments identified a major barrier in *A. fijiensis*: limited membrane permeability. This phenomenon has also been noted in *T. reesei*, where chemical facilitation was necessary for editing [31]. The marked increase in transformation efficiency after Triton X-100 treatment indicates that the dense cell wall–membrane structure physically obstructs the uptake of large Cas9–sgRNA complexes. While optimized RNP delivery alone yielded numerous transformants in *A. fijiensis*, sequencing revealed predominantly short deletions, confirming repair via NHEJ. When applying the RNP-with-donor strategy to *A. fijiensis*, we failed to obtain any insertion mutation transformants. In contrast to previous reports in *A. fumigatus* [34], *T. reesei* [31], and *Schizophyllum commune* [46], co-delivery of RNPs with donor DNA containing homology arms failed to produce transformants in our system, suggesting interspecies variation in repair pathway engagement following transient Cas9 activity. Published *Aspergillus* RNP-with-donor studies fall into at least two mechanistically distinct design classes. First, RNP + HDR workflows deliberately use long homology arms to bias repair toward Rad51-dependent homologous recombination; for example, in *A. niger*, correct donor insertion is described as HDR-mediated and requires ~1000–1500 bp flanks to sustain high targeting performance, whereas shortening flanks to 500 bp reduces both CFU and targeting efficiency and 100 bp yields no correct colonies [47]. Second, RNP + MMEJ workflows use short (≈35–50 bp) microhomology repair templates to engage MMEJ-mediated repair and can reach substantial KO efficiencies in *A. fumigatus* (e.g., 46–74% in wild-type background) [48]. Endogenous microhomology-mediated end joining is an intrinsic, error-prone double-strand break (DSB) repair route in filamentous fungi that anneals very short microhomologies flanking the break and typically produces small deletions/indels at the junction, thereby competing with canonical NHEJ and HDR and measurably shaping genome-editing outcomes [49]. In *A. fumigatus*, CRISPR-induced edits can be strongly biased toward MMEJ and remain efficient even when Ku-dependent NHEJ is genetically compromised, supporting the view that MMEJ is a robust endogenous “fallback” pathway that can be deliberately harnessed by appropriately designed microhomology donors [34]. Therefore, in *A. fijiensis*, the inability to recover transformants upon RNP + donor co-delivery is not merely a “lower efficiency” issue, but a qualitative barrier that prevents leveraging either HDR-long-arm or MMEJ-like microhomology template strategies reported in other *Aspergillus* species.

In our system, by contrast, implementing a microhomology-governed donor architecture (i.e., incorporation of correctly designed short microhomology arms) reprogrammed repair away from the default NHEJ mode and enabled high-efficiency, MMEJ-mediated editing. Quantitatively, this translated into a KO efficiency of 78% and a KI efficiency of 92.3% in *A. fijiensis*, which compares favorably to multiple published results, particularly for knock-in, where efficiencies are often the limiting step. In *A. nidulans*, MMEJ-based strategies using short microhomology have been summarized as achieving ~100% efficiency for small deletions and knock-in, reinforcing that MMEJ can rival or exceed HR while markedly simplifying donor construction [50]. Similar high-efficiency editing via MMEJ using short homology arms has been validated in *A. fumigatus* [34], suggesting that this pathway is a conserved yet not fully studied mechanism for genome engineering in Aspergilli. Conversely, precise knock-in can remain a practical bottleneck in some *Aspergillus* systems when pathway control is not optimized; for example, in *A. fumigatus*, pksP knock-out was reported at ~10.22%, while subsequent knock-in screens yielded only less than 10% correctly targeted GFP integrants and 23% eglA cassette candidates with expected bands, highlighting the difficulty of reliable insertion under conventional workflows [51]. However, unlike prior fungal MMEJ studies, our work systematically dissected the decisive role of homology sequence identity in pathway choice. We specifically demonstrated that for precise DNA fragment insertion via MMEJ, maintaining triple-sequence homology—between the two MHS flanking the Cas9-induced double-strand break in the genome and the corresponding 5 bp microhomology arms in the donor DNA—is critical. Furthermore, our results proved a strong preference for the MMEJ repair pathway under these conditions and identified that extending homologous arms beyond the core microhomology region significantly enhances editing efficiency. Taken together, these data position the *A. fijiensis* MMEJ framework as a high-throughput alternative to HDR in non-model *Aspergillus*, delivering KI efficiencies (92.3%) that exceed many HR-dependent or plasmid-only systems and approach the upper range of short-homology, MMEJ-enabled editing reported across the genus. Compared with HDR requiring long homology arms, short-microhomology MMEJ designs provide a route to improve both operational simplicity and targeting robustness without long HDR arms. The MMEJ approach significantly simplifies process complexity while preserving targeting specificity [20]. 

Although we established an effective RNP–MMEJ editing platform for *A. fijiensis*, several mechanistic questions remain unresolved. First, we did not perform a genome-wide assessment of off-target activity, which remains a limitation of the present study. Nevertheless, the sgRNAs used in this study were selected to minimize predicted off-target potential in the *A. fijiensis* genome, and few top-ranked candidate loci with putative miss-matches were prioritized for targeted validation. PCR amplification and Sanger sequencing of these predicted off-target sites in representative independent edited clones did not reveal detectable sequence alterations, supporting a high degree of editing specificity under our RNP delivery conditions. While these targeted checks still cannot exclude rare or structurally complex off-target events, off-target mutagenesis requires further investigation. Second, while long-fragment deletions and targeted insertions were achieved, the knock-in size limits and PAM-MHS distance dependency of these edits remain to be defined. Nonetheless, the framework developed here represents a critical step toward transforming *A. fijiensis* from a genetically recalcitrant organism into a tractable platform for functional genomics and secondary metabolite engineering.

## 5. Conclusions

In summary, this study established a practical and experimentally validated framework for genome manipulation in *A. fijiensis*, a previously genetically recalcitrant filamentous fungus. Through systematic optimization of protoplast preparation and comparative evaluation of plasmid-based and RNP-mediated CRISPR–Cas9 strategies, we demonstrated that conventional HDR-dependent approaches are inefficient in this species due to strong NHEJ activity. In contrast, precise exploitation of the endogenous MMEJ pathway using short, well-designed microhomology arms enabled highly efficient and reproducible genome editing. Our findings highlight the critical importance of species-specific optimization and reveal MMEJ as a dominant and tractable repair route in *A. fijiensis*. This RNP–MMEJ-based strategy substantially reduces construct complexity while maintaining targeting precision, providing a robust foundation for functional genomics and metabolic engineering in *A. fijiensis* and potentially other non-model filamentous fungi.

## Figures and Tables

**Table 1:** Components of different enzymatic combinations applied on the release of *A. fijiensis* protoplasts in every 10 mL digestion solution.

Combination	Lywallzyme	Yatalase	Cellulase	Snailase	Lysozyme	Driselase
C1	0.4 g	0.4 g				
C2	0.4 g					0.15 g
C3	0.4 g	0.4 g	0.6 g			
C4	0.4 g	0.4 g		0.1 g	0.1 g	
C5	0.4 g			0.1 g	0.1 g	
C6	0.4 g			0.1 g	0.1 g	0.15 g

**Table 2:** Influence of RNP dosage on transformation output and genome editing efficiency.

RNP (nM)	CFUs	Edited Clones	KO Efficiency (%)
0	0	-	-
20	0	-	-
50	6	2	33
100	18	9	50
150	42	23	55
200	55	43	78
300	28	12	43

**Table 3:** Effect of microhomology arm length on MMEJ-mediated integration outcomes.

MHS Length (bp)	CFUs	Positive Clones	Insertion Efficiency (%)
0 + 40	84	0	0
3 + 0	92	7	7.6
5 + 0	85	17	20.0
8 + 0	87	11	12.6
5 + 15	100	89	89.0
5 + 35	91	84	92.3
5 + 55	87	67	77.0

## Data Availability

The original contributions presented in this study are included in the article. Further inquiries can be directed to the corresponding author(s). The data presented in this study are openly available in National Center for Biotechnology Information at https://www.ncbi.nlm.nih.gov/ (accessed on 1 January 2025), Accession number SAMN05660285.

## Supplementary Materials

The following supporting information can be downloaded at: https://www.mdpi.com/article/10.3390/jof12030165/s1, Figure S1. Molecular identification and phylogenetic placement of the experimental *Aspergillus fijiensis* strain. (a) Representative ClustalW multiple-sequence alignment of three taxonomic markers—ITS, caM (calmodulin), and benA (β-tubulin)—comparing the experimental isolate with the reference strains *A. fijiensis* CBS 313.89 and Aspergillus aculeatinus CBS 121060 and Aspergillus aculeatus CBS 172.66. Only partial regions of each alignment are shown; variable sites are visible as mismatches among taxa. (b) Maximum-likelihood phylogenetic tree inferred from the concatenated ITS–caM–benA dataset with 10,000 bootstrap replicates, showing that the experimental isolate clusters within the *A. fijiensis* clade and is clearly separated from A. aculeatinus and A. aculeatus; Figure S2. A microscopic check of (a) mycelia at 18 hours post-inoculation; (b) protoplasts released from the *A. fijiensis* mycelia under 18 h culture after 3 h digestion; Figure S3. In vitro cleavage assay of Cas9 RNP complexes. Agarose gel electrophoresis showing the in vitro DNA cleavage activity of Cas9 under different assembly conditions. M, DNA size marker; WT, untreated target DNA; Lane 1, target DNA incubated with Cas9 protein in the absence of sgRNA; Lane 2, target DNA treated with preassembled Cas9–sgRNA RNP complex, displaying specific cleavage products; Table S1: List of primers; Table S2: Effect of PEG–Triton X-100 concentration on protoplast transformation efficiency.

## Abbreviations

The following abbreviations are used in this manuscript:

AMA1	Autonomous maintenance in *Aspergillus* element 1
CFU	Colony-forming units
DSB	Double-strand break
5-FOA	5-Fluoroorotic acid
HDR	Homology-directed repair
HPH	Hygromycin B phosphotransferase (hygromycin resistance gene)
MMEJ	Microhomology-mediated end joining
MHS	Microhomology sequence
NHEJ	Non-homologous end joining
RNP	Ribonucleoprotein
PAM	Protospacer adjacent motif
