# Supplementary material for: A High-Efficiency CRISPR–Cas9 Ribonucleoprotein Genome Editing System in Aspergillus fijiensis Enabled by Microhomology-Mediated End Joining
Source: J Fungi (Basel). 2026 Feb 25;12(3):165. doi: 10.3390/jof12030165 (PMC13027702; doi:10.3390/jof12030165)
Supplement: Supplementary file 1 [file jof-12-00165-s001.zip › jof-4113536-supplementary.pdf]

Supplementary Materials:

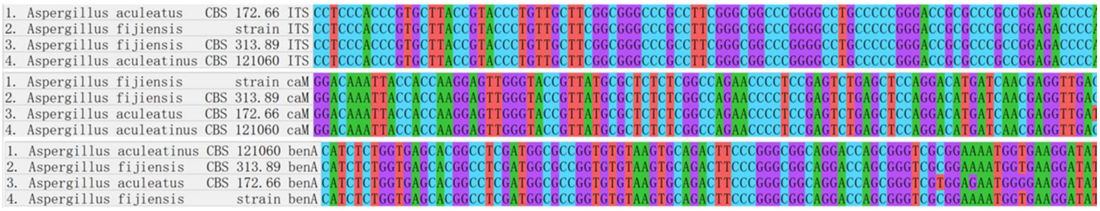

（a）

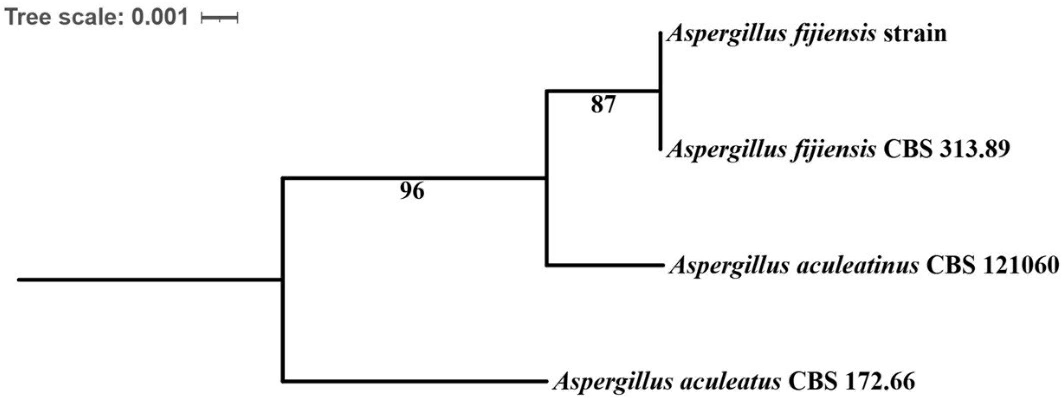

（b）

Figure S1. Molecular identification and phylogenetic placement of the experimental *Aspergillus fijiensis* strain. (a) Representative ClustalW multiple-sequence alignment of three taxonomic markers—*ITS*, *caM* (*calmodulin*), and *benA* (*β-tubulin*)—comparing the experimental isolate with the reference strains *A. fijiensis* CBS 313.89 and *Aspergillus aculeatinus* CBS 121060 and *Aspergillus aculeatus* CBS 172.66. Only partial regions of each alignment are shown; variable sites are visible as mismatches among taxa. (b) Maximum-likelihood phylogenetic tree inferred from the concatenated *ITS–caM–benA* dataset with 10,000 bootstrap replicates, showing that the experimental isolate clusters within the *A. fijiensis* clade and is clearly separated from *A. aculeatinus* and *A. aculeatus*.

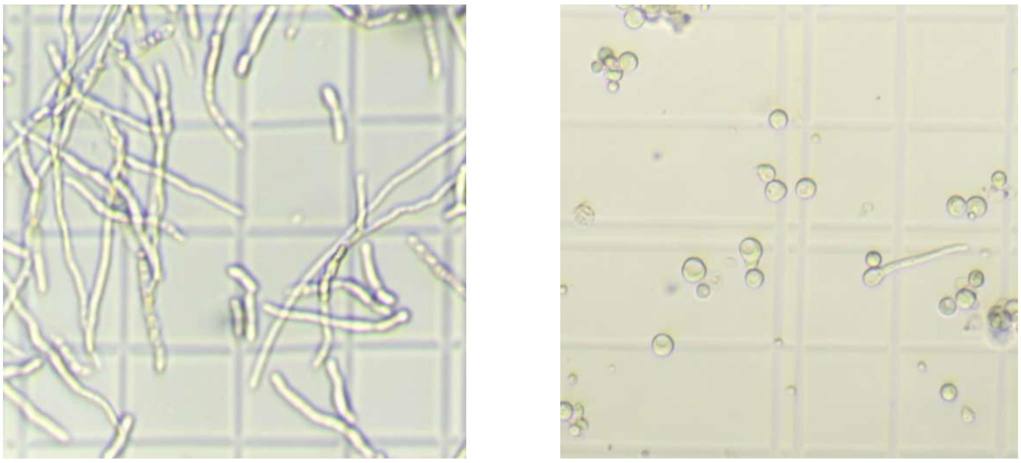

（a）（b）

Figure S2. A microscopic check of (a) Mycelia at 18 hours poet-inoculation; (b) protoplasts released from the *Aspergillus fijiensis* mycelia under 18 h culture after 3 h digestion, respectively.

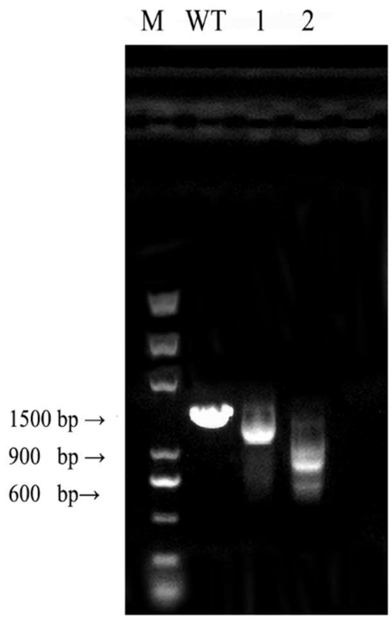

Figure S3. In vitro cleavage assay of Cas9 RNP complexes. Agarose gel electrophoresis showing the in vitro DNA cleavage activity of Cas9 under different assembly conditions. M, 100-5000 bp DNA Marker III (Biosharp BL103A，Anhui，China); WT, untreated target DNA; Lane 1, target DNA incubated with Cas9 protein in the absence of sgRNA; Lane 2, target DNA treated with preassembled Cas9–sgRNA ribonucleoprotein (RNP) complex, displaying specific cleavage products.

Table S1. List of primers

Name	Sequence (5' to 3')	Note
gRNA-scaffold-F	GGGTCTTCGAGAAGACCTGT	sgRNA scaffold cloning
gRNA-scaffold-R	AAAAAAGCACCGACTCGGTGCC	
U6-1-F	CTAGACTACCCCAAAGACGA	U6 promoter cloning
U6-1-R	ACAGGTCTTCTCGAAGACCCAGTTAC ATTTCAACAGATTA	
U6-*pyrG*-F	TGCAGTTGATGATGTGTTTTAGAGCTA GAAATAGCAAGTTAAAATAAG	sgRNA seamless cloning
U6-1-*pyrG*-R	CATCAACTGCAGCATCAGTTACATTTC AACAGATTA	
pAMA-1-F	TCGTCTTTGGGGTAGTCTAGGCACTTT TCGGGGAAATGTG	Vector backbone cloning
pAMA-R	CACCGAGTCGGTGCTTTTTTGTAAAAC GACGGCCAGTGAA	
D-F	TTCCCTGCAGGATCGAAACC	*pyrG* mutation detection primers
D-R	GGTTGTTGAGGGATTGGTGC	
hph-F	GATCTCGGAGTGGGCCCACAAAGTGG AAAGGCTGGTGTGC	Hygromycin (*hph*) gene cloning
hph-R	CAGGATGCTGCAGTTGATGATCGCGT GGAGCCAAGAGCGG	
5′flanking-*hph*-F	AGGGAGTTATCTGCGACCACTCGTCA CGGGCAGCACCAGG	Cloning of the 5' flanking region of the *pyrG* gene
5′flanking-*hph*-R	TGTGGGCCCACTCCGAGATC	
3′flanking-*hph*-F	TCATCAACTGCAGCATCCTG	PCloning of the 3' flanking region of the *pyrG* gene
3′flanking-*hph*-R	CAGCTCATCTGCAATGCATGCGGGTG TGACAGACAGGAGG	
T7-*pyrG*-1-F	TAATACGACTCACTATAGATGCTGCA GTTGATGATGTGTTTTAGAGCTAGA	In vitro synthesis of *pyrG* gene sgRNA
Donor-*hph*-F	TACCACAAGGGCACCCTCCGGATCTC GGAGTGGGCCCACAAAGTGGAAAGG CTGGTGTGC	Cloning of the *hph* insertion template
Donor-*hph*-R	TCCACGATGCCCTCGCCGGGCAGGAT GCTGCAGTTGATGATCGCGTGGAGCC AAGAGCGG	
T7-*pyrG*-2-F	TAATACGACTCACTATAGGGCCCACT CCGAGATCCGG GTTTTAGAGCTAGA	In vitro synthesis of *pyrG* gene sgRNA
Donor-MHS-*hph*-F	GATGCCCTCGCCGGGCAGGATGCTGC AGTTGATGATGTGGAAGTGGAAAGGC TGGTGTGC	Cloning of the *hph* insertion template with MHS
Donor-MHS-*hph*-R	TTCATCGACATCGGCAACACCGTCCA GAAGCAGTACCACATCGCGTGGAGCC AAGAGCGG	
T7-*pyrG*-3-F	TAATACGACTCACTATACTTGGTAGTG GTCACGTCGGGTTTTAGAGCTAGA	In vitro synthesis of *pyrG* gene sgRNA
T7-*pyrG*-4-F	TAATACGACTCACTATATCGCGGGAT CTATGCCGCGGGTTTTAGAGCTAGA	In vitro synthesis of *pyrG* gene sgRNA
Donor-3-*hph*-F	ACCAAGTGGAAAGGCTGGTGTGC	Cloning of the *hph* insertion template with 3-bp micro-
Donor-3-*hph*-R	GGTTCGCGTGGAGCCAAGAGCGG	

		homology sequence (MHS) arms
Donor-5-*hph*-F	AGAAGAAGTGGAAAGGCTGGTGTGC	Cloning of the *hph* insertion template with 5-bp micro-homology sequence (MHS) arms
Donor-5-*hph*-R	CTTCTTCGCGTGGAGCCAAGAGCGG	
Donor-7-*hph*-F	GCCCGTGAAGTGGAAAGGCTGGTGTG C	Cloning of the *hph* insertion template with 7-bp micro-homology sequence (MHS) arms
Donor-7-*hph*-R	CACGGGCTCGCGTGGAGCCAAGAGC GG	
Donor-5-15-*hph*-F	AGATCGCCGAGGCCAAGAAGAAGTG GAAAGGCTGGTGTGC	Cloning of the insertion template with 15-bp extended micro-homology arms
Donor-5-15-*hph*-R	CGCCTCCCAGCCCTCCTTCTTCGCGTG GAGCCAAGAGCGG	
Donor-5-25-*hph*-F	CGCCTCTTCGAGATCGCCGAGGCCAA GAAGAAGTGGAAAGGCTGGTGTGC	Cloning of the insertion template with 15-bp extended micro-homology arms
Donor-5-25-*hph*-R	GGGCCTGGTACGCCTCCCAGCCCTCC TTCTTCGCGTGGAGCCAAGAGCGG	
Donor-5-35-*hph*-F	TCTGGCCAAGCGCCTCTTCGAGATCG CCGAGGCCAAGAAG AAGTGGAAAGGCTGGTGTGC	Cloning of the insertion template with 15-bp extended micro-homology arms
Donor-5-35-*hph*-R	CCGCCAACACGGGCCTGGTACGCCTC CCAGCCCTCCTTCT TCGCGTGGAGCCAAGAGCGG	
Donor-*hph*-d-F	TAATTGGCCCATCCGGCATC	Detection of *hph* gene insertion
Donor-*hph*-d-R	TATTCCTTTGCCCTCGGACG	

Table S2. Effect of PEG–Triton X-100 concentration on protoplast transformation efficiency

Condition	CFUs	Relative increase
55% PEG	55	1.0
55% PEG + Triton	85	1.5×
